# Subhepatic Appendicitis in an 11-year-old Boy: A Case Report

**DOI:** 10.7759/cureus.6489

**Published:** 2019-12-28

**Authors:** Saad M Alqahtani, Mohamed Lasheen, Showkat Paray

**Affiliations:** 1 Department of Surgery, College of Medicine, Majmaah University, Majmaah, SAU; 2 Department of Surgery, King Khalid General Hospital, Majmaah, SAU

**Keywords:** subhepatic appendix, appendicitis, case report, pediatric

## Abstract

Appendicitis is a common surgical emergency. When present in an abnormal subhepatic location, it can pose a challenge in its diagnosis and management. Subhepatic appendicitis is a rare phenomenon, especially in the pediatric age group. Herein, we present a rare case of subhepatic appendicitis in an 11-year-old boy with delayed presentation but managed successfully.

## Introduction

The appendix is a small structure that belongs to the digestive tract system. Its most common positions are the pelvic cavity as well as the descending intraperitoneal and retrocecal areas. The appendix is very vulnerable to acute inflammatory processes, and acute appendicitis continues to be one of the most frequently encountered surgical emergencies in pediatrics and adults. The site of normally placed appendix and its classical presentation of appendicitis are well documented in the literature. However, the variations in the anatomical position of the appendix contribute to the difficulty in diagnosing appendicitis [1]. 

Appendicitis taking place in the subhepatic space (i.e., subhepatic appendicitis) is largely not common, and it occurs as a result of intestinal malrotation and/or non-decent of the cecum during embryonic development [2]. Owing to its unusual site, subhepatic appendicitis presents a substantial challenge in its diagnosis and management as it can mimic upper abdominal conditions [2].

Unusual presentations of appendicitis (generally) and subhepatic appendicitis (specifically) lead to a delayed diagnosis that contributes to an increase in morbidity [3].

## Case presentation

An 11-year-old boy, otherwise healthy, presented to emergency department with an acute onset of upper abdominal pain experienced for one day. The pain was associated with few episodes of vomiting, fever and constipation. The patient was treated symptomatically in a nearby hospital and discharged. Three days later, he presented to our hospital with moderate abdominal pain in the right upper quadrant and the right flank areas. The pain was continuous, non-radiating and associated with mild dysuria and fever.

On clinical examination, the patient was mildly dehydrated. He had a body temperature of 38.8 degrees Celsius and his pulse was 112 beats/min. The right hypochondrium and right lumbar areas were tender, with minimal tenderness in the other quadrants. No guarding or rigidity was detected.

**Figure 1:**
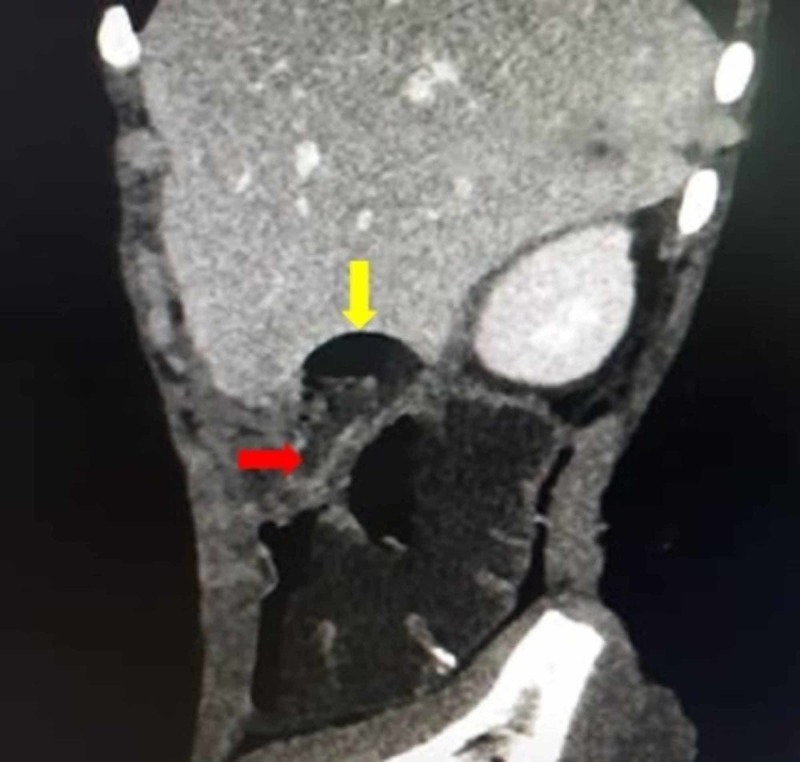
Sagittal CT scan of the abdomen depicting a dilated inflamed appendix with a perforated tip and an air pocket. The yellow arrow shows the perforated tip of the appendix with collection. The red arrow shows the base of the appendix.

**Figure 2:**
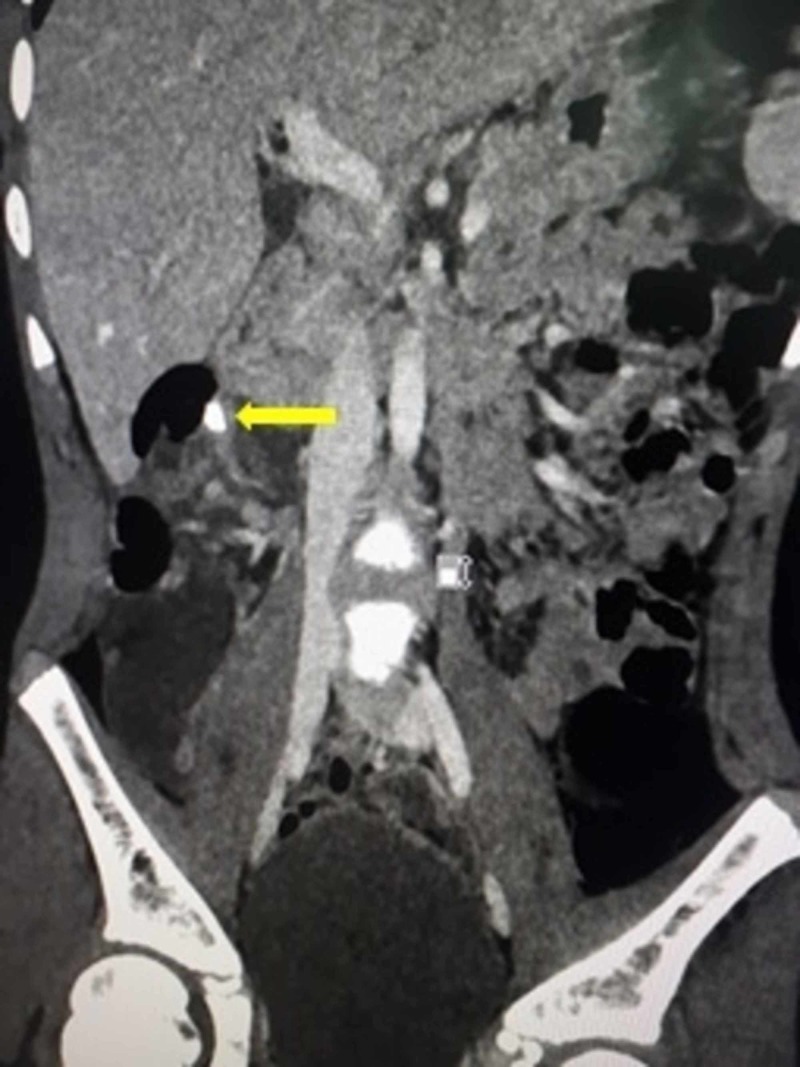
Coronoal CT scan of the abdomen depicting a subhepatic appendicolith (yellow arrow).

On initial work-up, the complete blood count showed leukocytosis-a white blood count of 18 x 10^9^ cells/L. All other investigations were normal. Ultrasonography of the abdomen revealed a collection in the subhepatic area in relation to the right kidney enabling a provisional diagnosis of a perinephric abscess. After admission and initial management with intravenous (IV) fluids and antibiotics, the patient was advised a computed tomography (CT) scan of the abdomen with IV contrast. The CT scan revealed a well-defined tubular structure lateral to the colon in the right hypochondrium with fat stranding extending cranially from its base. A 4 x 3 x 2 cm collection was detected in Morison's pouch indenting the edge of the liver. There were free air pockets associated with the collection (Figures 1, 2).

**Figure 3:**
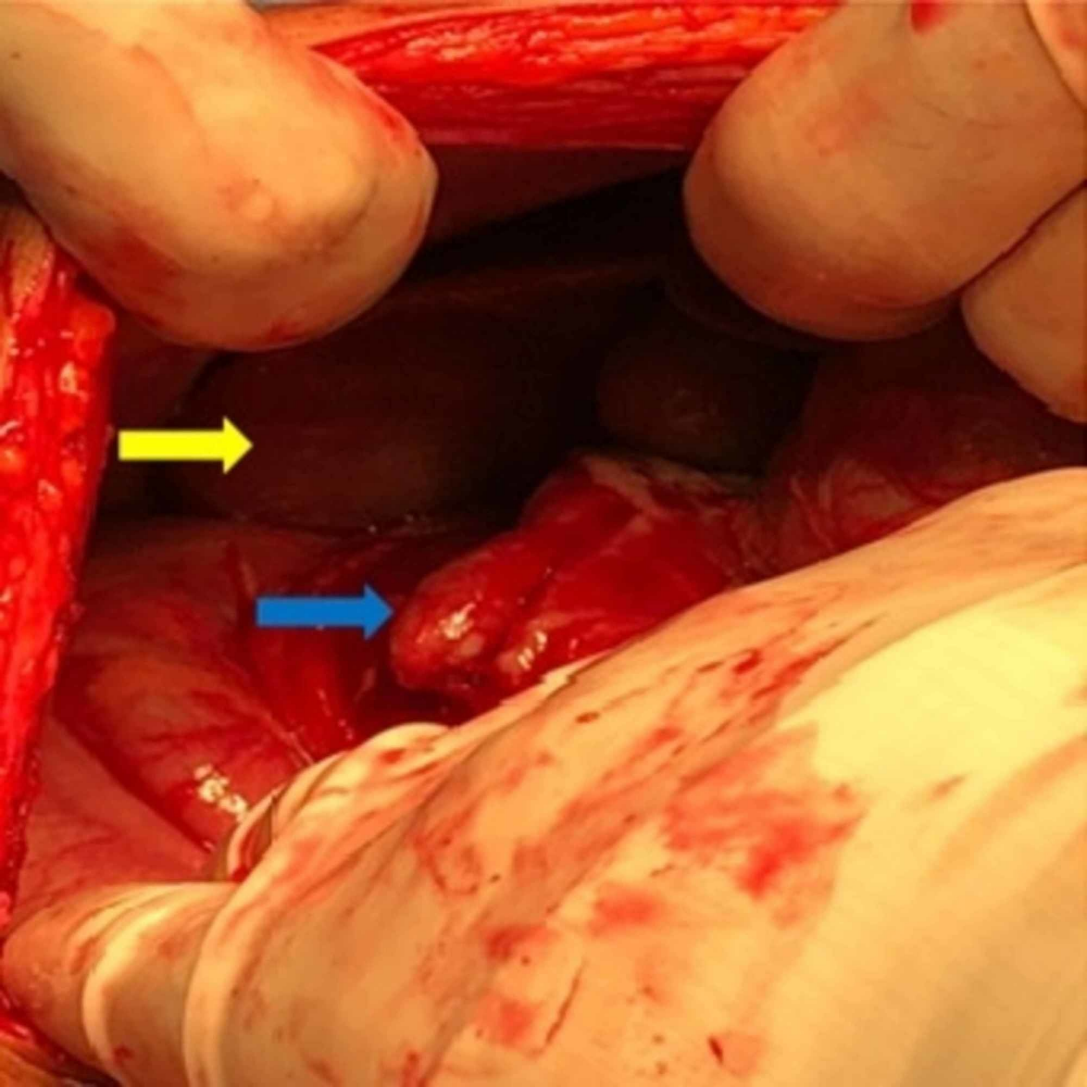
Intraoperative image showing the subhepatic appendix (blue arrow) and the liver (yellow arrow).

A diagnosis of an abnormally located perforated appendix with localized collection was made, and the patient was subjected to laparotomy. During exploration, an abnormally high lying cecum was observed in the right hypochondrium; the ascending colon was absent and a redundant dilated transverse colon was seen. An early inflammatory mass involving the cecum, transverse colon, omentum and the duodenum was detected. Upon manipulation, a pocket of pus was detected and drained (approximately 20 ml). The cecum was mobilized by incising the lateral peritoneal reflection. The appendix was located immediately medial to the ileocecal junction craniocaudally with the tip trailing superiorly in Morison's pouch. The appendix was grossly inflamed with an unhealthy base (Figure 3). The terminal ileum was abnormally retroperitoneal, winding around the cecum with the ileocecal junction on the anterolateral aspect. Appendectomy was performed and followed by proper lavage and placement of drain.

The patient had an uneventful recovery and was discharged home on the fifth postoperative day. The patient was seen in the outpatient clinic, and he was in his usual state of health.

## Discussion

Acute appendicitis is the most common surgical emergency in children. The most common location of the appendix is retrocecal (65.3%). However, other rare positions have been reported including subhepatic, lateral pouch, mesoceliac, left-sided, intraherniary and lumbar [4,5]. When the appendix is located in an atypical position, patients may present with unusual clinical symptoms. This can result in a delay in the diagnosis and unfavorable complications such as perforation and abscess formation [1,6].

In 1955, King described the ﬁrst case of subhepatic appendicitis and concluded that in such conditions the cecum remains in the subhepatic area [7]. Subhepatic appendicitis is a rare disease and accounts for 0.08% of all cases of appendicitis with a marginally higher incidence of 1% and 3.2% [3,5,8] in other studies. Although the majority of reports mentioned in the literature discuss the incidence of subhepatic appendicitis in adults, only a few cases have been reported in the pediatric age group [2,3,8-11].

While an ultrasound scan of the abdomen is the first radiological investigation, it has a high probability of misdiagnosis [12]. Nevertheless, a CT scan is the best modality to identify subhepatic appendicitis [2] as it is reported to have high sensitivity (100%), specificity (95%) and accuracy (98%) in establishing the diagnosis of acute appendicitis [13].

A controversy remains regarding the best approach for cases of complicated appendicitis in children [3]. Broadly speaking, there are two main surgical approaches, namely open and laparoscopic appendectomy. Accumulating evidence from earlier studies demonstrated superiority for the laparoscopic appendectomy over the open appendectomy [3,5,14]. The laparoscopic appendectomy approach yields numerous advantages, such as reduced incidence of postoperative ileus, lower frequency of surgical wound infection, shorter duration of hospitalization, lesser utility of postoperative pain killers, decreased occurrence of adhesions, minimal body mutilation and faster recovery to previous status of well-being [14]. We, the authors, are in favor of the laparoscopic appendectomy approach, too. However, the reason for not performing a laparoscopic appendectomy in our case was attributable to the lack of laparoscopic facility in our healthcare center. An additional and important parameter of success in performing laparoscopic appendectomy is the expertise of the operating surgeon. 

## Conclusions

Acute subhepatic appendicitis is a very rare condition and difficult to treat. Its unusual location may delay and complicate the underlying pathology. However, a high degree of clinical suspicion and superior imaging modalities like CT scan of the abdomen can aid in the diagnosis and appropriate timely intervention.
